# An isolated vaginal metastasis from intestinal signet ring cell carcinoma: a case report and literature review

**DOI:** 10.1186/s12885-020-06950-x

**Published:** 2020-05-27

**Authors:** Xiao Dan Zhu, Jin Wang, Qin Han You, Tian An Jiang

**Affiliations:** The First Affiliated Hospital of Zhejiang University School of Medicine, Hangzhou, China

**Keywords:** Vaginal metastasis, Intestinal signet ring cell carcinoma, Vaginal chronic inflammation, Ultrasound

## Abstract

**Background:**

Isolated vaginal metastases from intestinal signet ring cell carcinoma are extremely rare. There are no reported cases in the domestic or foreign literature. The characteristics of such cases of metastasis remain relatively unknown. As a life-threatening malignant tumor, it is very important to carry out a systemic tumor examination and transvaginal biopsy, even though clinical symptoms are not typical and there is no systemic tumor history.

**Case presentation:**

We present a case of an isolated vaginal metastasis from intestinal cancer in a 45-year-old female patient. The patient experienced a small amount of irregular vaginal bleeding and difficulty urinating. She had no history of systemic cancer. An early physical examination and transvaginal ultrasound (TVS) showed marked thickening of the entire vaginal wall. Pelvic nuclear magnetic resonance imaging (MRI) and a colposcopic biopsy were used to diagnose her with chronic vaginitis. An analysis of the vaginal wall biopsy showed signet ring cell carcinoma. Colorectal colonoscopy revealed advanced interstitial signet ring cell carcinoma as the primary source of vaginal wall infiltration. We review previous case reports of vaginal metastases from colorectal cancer and discuss the symptoms, pathological type, and outcomes.

**Conclusions:**

We hypothesize that vaginal wall thickening and stiffness accompanied by chronic inflammatory-like changes may be clinical features of a vaginal metastasis of signet ring cell carcinoma of the intestine. We also emphasize that it is very important to perform a systemic tumor examination in a timely manner when a patient has the abovementioned symptoms.

## Background

Isolated vaginal metastases from intestinal signet ring cell carcinoma are very rare entities and have not been reported in the literature thus far. We searched PubMed, Medline and EMBASE to identify all articles published in the English language after 1960 and before Dec 31, 2018, pertaining to vaginal metastases from intestinal signet ring cell carcinoma. There are only a few previous reports of vaginal metastases from colorectal cancer in the literature, and the pathological type was not signet ring cell carcinoma [1]. Most of these patients usually had other metastatic lesions in locations such as the liver or breast. It is very difficult to diagnose a vaginal metastasis when the patient has no history of systemic tumors and no significant vaginal mass. In addition, the characteristics of such cases of metastasis remain relatively unknown. In this report, we highlight the importance and necessity of performing a systemic tumor examination when patients have symptoms similar to those of chronic vaginal inflammation and that match the clinical features of a vaginal metastasis of signet ring cell carcinoma of the intestine.

## Case presentation

**Fig. 1:**
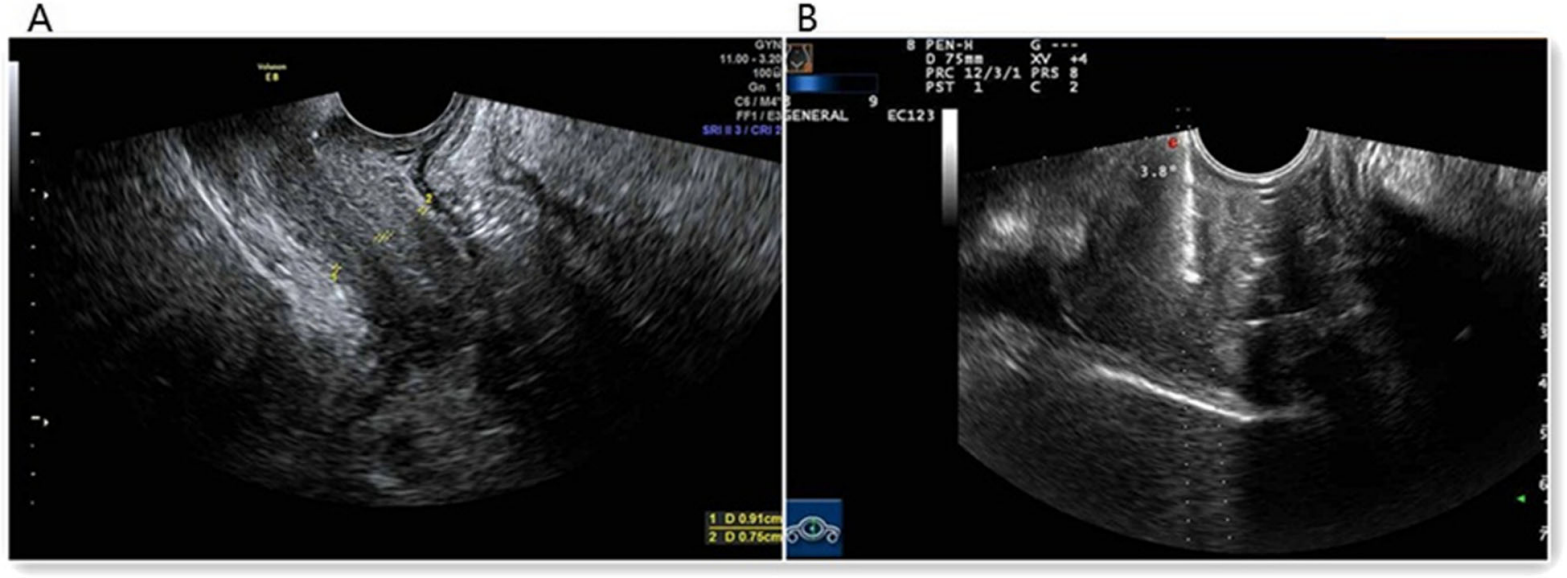
Ultrasound examination image. **a**.: TVS showed clear uniform thickening of the vaginal wall. **b**: TVS-guided vaginal wall biopsy

**Fig. 2:**
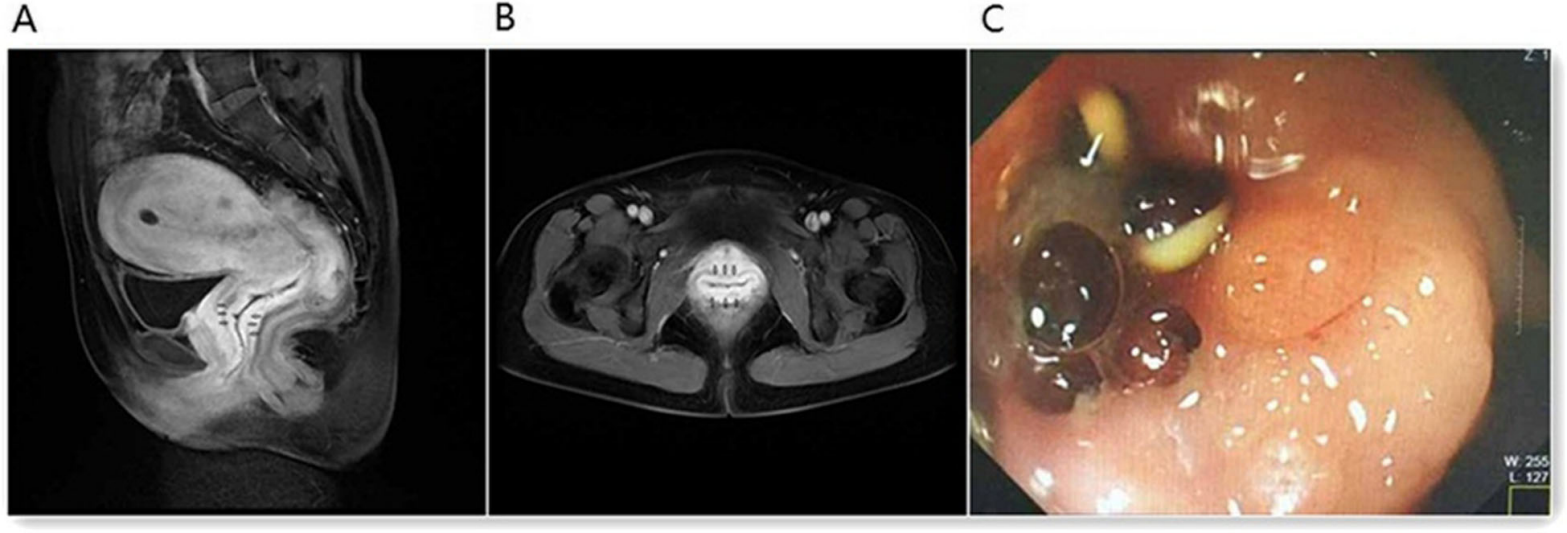
MRI and colorectal colonoscopy. **a** and **b**: Pelvic MRI showed significant thickening of the vaginal wall with enhancement. **c**: Colorectal colonoscopy revealed multiple lesions in the ileocecal valve and rectum

**Fig. 3:**
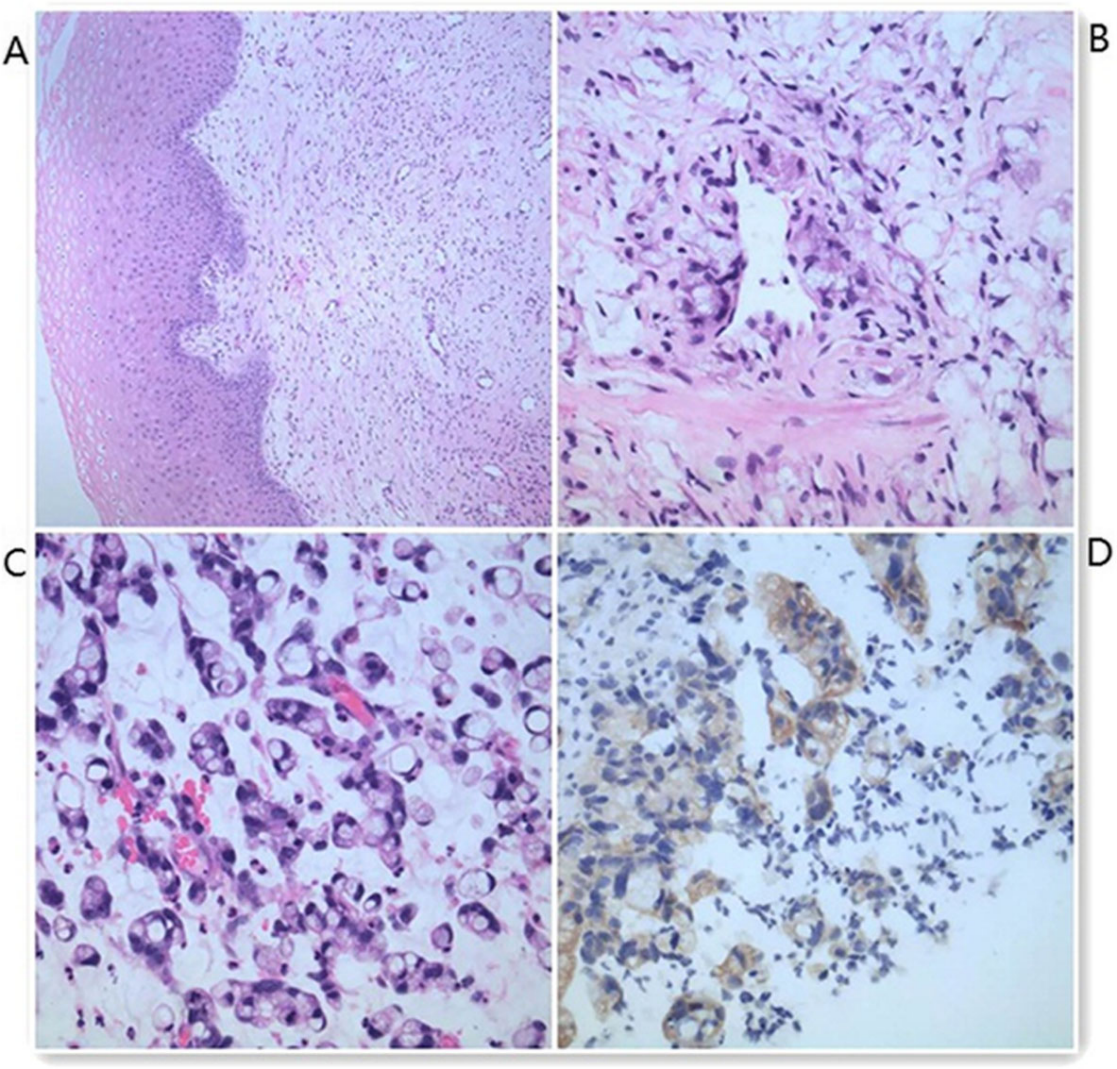
Pathological examination. **a**: Colposcopic biopsy: Microscopic hematoxylin-eosin stained section with an original magnification of 100 showed squamos epithelium with a few lymphocytes infiltrating the stroma. **b**: TVS-guided vaginal wall biopsy: A microscopic hematoxylin-eosin-stained section with an original magnification of 400 showed adenocarcinoma cells that contained considerable mucus with a nucleus pushed into a crescent shape. **c**: Colorectal colonoscopy: A microscopic hematoxylin-eosin-stained section with an original magnification of 400 (ileocecal valve and rectal) showed adenocarcinoma cells that contain considerable mucus with a nucleus pushed into a cresent shape. **d**: Immunohistochemistry showed neoplastic cells that stained positive for CK

A 45-year-old Chinese woman visited our hospital with a small amount of irregular vaginal bleeding and difficulty urinating. The patient had no history of systemic cancer, malignant lymphoma, or any gastrointestinal discomfort. A previous medical examination report was normal. Her family history was also unremarkable. During gynecological examinations, the gynecologist found vaginal stiffness similar to that observed in a frozen pelvis. When the patient underwent the first transvaginal ultrasound (TVS), the sonographer felt that the patient’s vaginal wall was very stiff. The probe had a significant obstruction when entering the vagina, and it could not completely enter the vagina. TVS showed a marked thickening of the entire vaginal wall, with an anterior wall thickness of approximately 0.91 cm and a posterior wall thickness of approximately 0.75 cm (Fig. 1a). In addition, the patient had no obvious abnormal signs in the cervix or vagina. Pelvic magnetic resonance imaging (MRI) showed vaginal wall thickening with obvious enhancement and multiple lymph nodes visible in the pelvic cavity. MRI showed chronic inflammation (Fig. 2a and b). Cervical ThinPrep cytology results were normal. Other laboratory tests including tumor marker levels (alpha fetoprotein: 1.9 ng/ml, carcinoembryonic antigen: 4.0 ng/ml, cancer antigen 125 II: 19.0 U/ml, cancer antigen 199XF: 12.0 U/ml, ferritin: 128.8 ng/ml, cancer antigen 153: 16.5 U/ml, serum chorionic gonadotropin: < 0.6 IU/ml, squamous cell carcinoma antigen: 0.8 ng/ml) and sex hormone indices (testosterone: 30.9 ng/dl, estradiol: 52.8 pg/ml, follicle-stimulating hormone: 6.4 mlU/ml, luteinizing hormone: 1.7 mlU/ml, prolactin: 19.4 ng/ml, progesterone < 0.21 ng/ml) were within the normal ranges. The patient then underwent a colposcopic biopsy, and the pathology suggested chronic inflammation of the mucosa with interstitial edema (Fig. 3a). She was initially diagnosed with chronic vaginitis and received anti-inflammatory treatment for 2 weeks.

After 2 weeks, the same sonographer performed another TVS and felt that the patient’s vaginal wall stiffness and obstruction were significantly better than before. The probe could enter the vagina completely. The scan results were basically the same as the previous results, and the vaginal wall was still very thick. After the scan, there were many sticky secretions flowing out of the vagina. The patient underwent a TVS-guided vaginal wall biopsy at that time (Fig. 1b). Pathological results suggested ring-like cell infiltration in the fibrous tissue, suggesting that the primary lesion may be derived from the stomach or intestine (Fig. 3b). Colorectal colonoscopy revealed multiple ileocecal valve and rectal lesions (Fig. 2c). Pathological results suggested diffuse infiltration of signet-like cells in the mucosa of the ileocecal valve and rectum suggestive of signet ring cell carcinoma (Fig. 3c and d). The monoclonal antibodies and oncogenes used for detection were as follows: cytokeratin (CK(+)), epithelial membrane antigen (EMA(+)), cluster of differentiation 68(CD68(−)), human mutL homolog1 (hMLH1(+)), human mutS homolog2(hMSH2(+)), human mutS homolog 6(hMSH6(+)), and postmeiotic segregation increased 2 (PMS2(+)). To date, the patient has received a clear diagnosis: signet ring cell carcinoma originating in the intestine with a vaginal metastasis. The clinical staging is IVa. Because the patient did not receive KRAS and BRAF gene tests, we cannot further analyze the mutation status. No other metastases were found. Unfortunately, the patient gave up treatment.

## Discussion and conclusions

Of gynecological malignancies, primary vaginal tumors account for only 1%, and the pathological type is mainly squamous cell carcinoma [2]. Among vaginal metastases, the primary lesions are derived mainly from the uterus [3] and rarely from the colon, rectum, kidney, breast and pancreas. The primary tumor in this case was derived from a vaginal metastasis of a colorectal lesion, and the pathological type was basically adenocarcinoma [4, 5]. We reviewed the literature and found that metastatic lesions of gastrointestinal signet ring cell carcinoma and adenocarcinoma involve the breast, testis, iris, cervix, and myometrium [6–10]. There are no reports of a vaginal metastasis of signet ring cell carcinoma in the gastrointestinal tract. We reviewed the case reports of vaginal metastases of colorectal cancer from 1953 to 2018 domestically and abroad and found that most cases of vaginal metastases are accompanied by other organ metastases, such as those in the lungs, liver, and bones. Sadatomo A [1] conducted a literature review of all English cases of isolated vaginal metastases from colorectal cancer from 1956 to 2015; there were only 10 isolated vaginal metastases from colorectal cancer (Table 1) [1, 11–15, 17, 18]. In addition, China reported a case of an isolated vaginal metastasis of rectal adenocarcinoma in 2010 [16]. The case we reported here is an isolated vaginal metastasis of colorectal cancer. In the previous cases, all pathological types were adenocarcinomas, and only our case was signet ring cell carcinoma.

**Table 1:** Cases of isolated vaginal metastasis from colorectal cancer

Author	Year	Age	complaint location	Vagina mass	Primary tumor	Pathology	Metastasis time	Outcome
Raider [11]	1966	63	Bleeding	Yes	Descending colon	Adenocarcinoma	2 year after primay operation	Alive for 4 years after vaginal recurrence
Lee SM [12]	1974	81	None	Yes	Sigmoid colon	Adenocarcinoma	Synchronous	Alive for 12 months after diagnosis
		57	None	Yes	Sigmoid colon	Adenocarcinoma	18 months after primay operation	Vaginal recurrence 1 year after diagnosis
Marchal F [13]	2006	81	Bleeding	Yes	Sigmoid colon	Adenocarcinoma	Synchronous	Alive for 39 months after diagnosis
Costa SRP [14]	2009	67	Bleeding	Yes	Right colon	Adenocarcinoma	3 months after primay operation	Alive for 4 years after diagnosis
Funada T [15]	2010	63	Perinea discomfort	Yes	Rectum	Adenocarcinoma	Synchronous	Alive for 1 years after diagnosis
Yin [16]	2010	68	Bleeding	Yes	Rectum	Adenocarcinoma	Synchronous	None
Sabbagh C [17]	2011	62	Bleeding	Yes	Rectum	Adenocarcinoma	Synchronous	Alive for 1 years after diagnosis
		78	None	Yes	Rectum	Adenocarcinoma	Synchronous	Alive for 1O months after surgery
D’Arco F [18]	2014	67	Bleeding	Yes	Sigmoid colon	Adenocarcinoma	Synchronous	None
Sadatomo [1]	2015	71	None	Yes	Rectum	Adenocarcinoma	Synchronous	Alive for 3 months after the recurrent tumor
Present case		45	Bleeding and urinary difficulty	No	Ileocecal valve and Rectum	Signet ring cell carcinoma	Synchronous	Abandon treatment

The clinical manifestations of vaginal metastases are mainly vaginal masses and vaginal bleeding, followed by vaginal fluid, vaginal staining or perineal discomfort [3]. Among the 11 patients with isolated vaginal metastases of colorectal adenocarcinoma, 5 complained of vaginal bleeding, 1 experienced perineal discomfort, 2 had no obvious symptoms, and 2 had unclear symptoms. Although nearly 50% of the patients complained of vaginal bleeding, we found vaginal masses in all 11 patients. Therefore, the corresponding examination can be used for a quick diagnosis, and it is difficult to miss the diagnosis or delay the diagnosis. In our case, the primary lesion did not have any clinical manifestations. The patient complained of a small amount of irregular vaginal bleeding and difficulty urinating. However, no vaginal mass was found on TVS, MRI or colposcopic biopsy. Studies have shown that MRI is very useful for assessing vaginal lesions and distinguishing between adenocarcinoma and squamous cell carcinoma [18]. However, this case showed thickening of the vaginal wall on MRI with obvious enhancement, suggesting only chronic inflammation of the vagina. The colposcopic biopsy suggested chronic mucosal inflammation with interstitial edema. So far, we have found that the clinical manifestations of an isolated vaginal metastasis of colorectal signet ring cell carcinoma and an isolated vaginal metastasis of colorectal adenocarcinoma are very different. In this case, the clinical manifestations and examinations of the patient were mainly based on chronic inflammatory changes, and even after 2 weeks of anti-inflammatory treatment, the symptoms of vaginal wall stiffness were greatly alleviated, which is undoubtedly very confusing. Under these conditions, if there is no obvious vaginal mass or lesion, it is difficult to provide a quick diagnosis. If the patient does not undergo a transvaginal vaginal wall biopsy, there is no doubt she will continue to experience a delayed diagnosis.

As there are no previous related case reports to use as a reference, according to the various clinical manifestations and test results in this case, we speculate that for intestinal signet ring cell carcinoma with a vaginal metastasis, thickening and stiffness of the vaginal wall accompanied by chronic inflammatory symptoms of the vagina may be important clinical manifestations. At the same time, we emphasize that when such patients are encountered, even if the patient does not have a history of a tumor or symptoms, a timely systemic tumor examination is still very important and necessary. Finally, we recommend that when diseased tissue needs to be taken for a biopsy due to stiffness of the vaginal wall and chronic inflammatory changes, a transvaginal vaginal wall biopsy undoubtedly has a greater advantage than a superficial colposcopic biopsy, as it is clearly difficult to obtain a satisfactory amount of diseased tissue.

## Data Availability

All the data supporting our findings are contained within the manuscript.

## Abbreviations

MRI: Magnetic resonance imaging
TVS: Transvaginal ultrasound
CK: Cytokeratin
EMA: Epithelial membrane antigen
CD: Cluster of differentiation
hMLH: human mutL homolog
hMSH: human mutS homolog
PMS2: Postmeiotic segregation increased 2
